# A Deep Learning Biomimetic Milky Way Compass

**DOI:** 10.3390/biomimetics9100620

**Published:** 2024-10-12

**Authors:** Yiting Tao, Michael Lucas, Asanka Perera, Samuel Teague, Timothy McIntyre, Titilayo Ogunwa, Eric Warrant, Javaan Chahl

**Affiliations:** 1 School of Engineering, University of South Australia, Mawson Lakes, SA 5095, Australia; michael.lucas@unisa.edu.au (M.L.); samuel.teague@mymail.unisa.edu.au (S.T.); timothy.mcintyre@unisa.edu.au (T.M.); titilayo.ogunwa@unisa.edu.au (T.O.); javaan.chahl@unisa.edu.au (J.C.); 2 School of Engineering, University of Southern Queensland, Springfield, QLD 4300, Australia; asanka.perera@unisq.edu.au; 3 Platforms Division, Defence Science and Technology Group, Edinburgh, SA 5095, Australia; 4 Lund Vision Group, Department of Biology, University of Lund, 22100 Lund, Sweden; eric.warrant@biol.lu.se

**Keywords:** biomimetic, Milky Way, YOLOv8, instance segmentation, orientation

## Abstract

Moving in straight lines is a behaviour that enables organisms to search for food, move away from threats, and ultimately seek suitable environments in which to survive and reproduce. This study explores a vision-based technique for detecting a change in heading direction using the Milky Way (MW), one of the navigational cues that are known to be used by night-active insects. An algorithm is proposed that combines the YOLOv8m-seg model and normalised second central moments to calculate the MW orientation angle. This method addresses many likely scenarios where segmentation of the MW from the background by image thresholding or edge detection is not applicable, such as when the moon is substantial or when anthropogenic light is present. The proposed YOLOv8m-seg model achieves a segment mAP@0.5 of 84.7% on the validation dataset using our own training dataset of MW images. To explore its potential role in autonomous system applications, we compare night sky imagery and GPS heading data from a field trial in rural South Australia. The comparison results show that for short-term navigation, the segmented MW image can be used as a reliable orientation cue. There is a difference of roughly 5–10° between the proposed method and GT as the path involves left or right 90° turns at certain locations.

## 1. Introduction

Animals have evolved to be adapted to the environmental and ecological conditions present on Earth. Their navigational sensory systems and behaviours are specifically tuned to cues in the environment that can lead them to food or shelter or away from noxious conditions or situations. Navigation is a fundamental behaviour for which there are a great diversity of biological solutions, all based on sensory information available on our planet.

In this paper, we explore orientation using the Milky Way (MW), one of the navigational cues used by night-active insects. Celestial navigation is used by technological systems, although they have tended to rely on tracking individual stars and clusters of stars. The MW is a different type of cue, visually represented as a band of stars and light spanning almost a complete arc in the southern night sky. This difference in the appearance of the MW compared to the positions of individual stars makes it interesting because it might allow alternate or simpler imaging systems to be deployed on autonomous platforms.

The MW is a difficult visual target, and its appearance varies with latitude and time of year. It is also prone to being washed out by light pollution, and it often has very low contrast due to atmospheric effects. In addition, it is not visible during the day, and is only partially visible in the Northern Hemisphere. On other planets, in orbit, and in interplanetary space, the MW is likely to be substantially more visible, so this navigational cue might be more useful well above Earth than on it.

This study aims to use YOLOv8 object detection techniques to establish a viable technological MW orientation detector, with the ultimate aim being to produce a viable instrument. The use of YOLOv8 technology also produces the opportunity to explore how the characteristics of the MW are encoded by the learning algorithm, potentially providing insight into detection architectures that might exist within the insect brain. It is hoped that in the future, this method will provide insight into the nature of simulated neural solutions to real-world sensory challenges.

## 2. Background

### 2.1. Celestial Navigation by Insects and Other Animals

From the most primitive organisms expressing phototaxis to organisms that build nests and hives, navigation is a critical behaviour. At the lowest level, navigation might mean the ability to travel in straight lines for a limited period of time. At higher levels, pinpoint localisation might be required, possibly across hundreds or thousands of kilometers. Straight-line navigation might be required to avoid retracing areas that have no resources or to move away from threats or competitors. Animals have been shown to exploit a number of sensory cues, including visual landmarks, Earth’s magnetic field, chemical plumes, wind, or odometry, to steer their locomotion. Celestial cues have been shown to be used by some species, including the position of the sun, the moon, and the orientation of the lunar or solar polarisation pattern [1,2,3]. The MW is a special case amongst the celestial options for orientation, since the method relies on the location of stars. The large extent of the MW and comparatively low contrast makes it usable for low-resolution but high-sensitivity visual systems, unlike individual stars.

**Figure 1:**
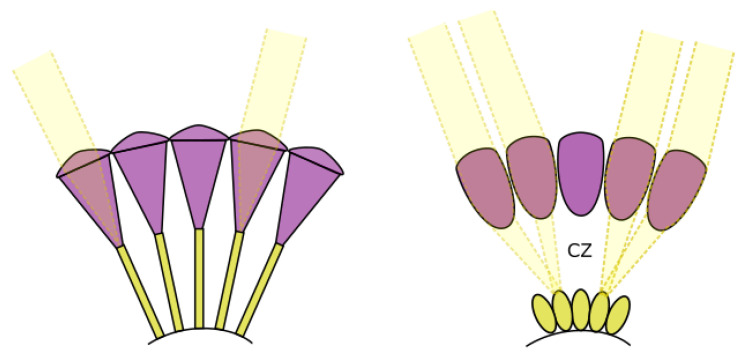
**Left**: apposition compound eyes; **right**: superposition compound eyes. The clear zone (CZ) is labelled in superposition eyes. Figure adapted from Warrant et al. [14].

Although the behaviour of insects is the inspiration for this study, insect visual perception is substantially different from that of humans. It operates in a very different manner than our visual system, and from the optics and electronics of conventional cameras. Insects have evolved compound eyes that use extraordinary adaptations and specialisations to meet the sensory requirements of operating in their environments [4,5]. The ability of insects to resolve details under starlit conditions is the first challenge they face. Their minute faceted optics and eye structures have adapted to capture enough light and preserve enough detail to allow insects to operate at night. The means by which this has been achieved is illustrated by examining two types of compound eyes, shown in Figure 1, apposition eyes and superposition eyes. In an apposition eye, each ommatidium is sheathed with light-absorbing screening pigment, which prevents light from spilling over to photoreceptors in neighboring ommatidia, ensuring that each photoreceptor only receives light from its corresponding lens. Apposition compound eyes are typically found in diurnal insects, which have evolved to achieve high spatial resolution when light is sufficient, such as dragonflies [6] and honeybees [7]. In superposition compound eyes, lenses are not associated with specific photoreceptors, so light is accepted from possibly hundreds of lenses, which improves light capture but reduces spatial resolution. This type of eye is commonly seen in insects that are active at night and in low-light conditions [2,8]. Also, the visual system of nocturnal insects typically engages in significant neural pooling through lateral neural interconnections [9]. The critical observation that comes from this is that the geometry and photoreceptor density of the eye does not define the spatial resolution, which is likely to be lower than the external optical geometry may indicate.

The sky’s polarisation pattern and its use by biological systems has been widely observed and researched in various species as the daytime celestial navigation cue [10,11]. With the aid of anatomical adaptations, some visual systems are able to perceive a clear blue sky as a sizable polarisation pattern that is aligned with the sun’s relative angular location. This perception is beneficial for a flying insect; without it, the sky would offer minimal directional information, whether absolute or relative, especially when the sun is close to the zenith, hidden by ground features, or blocked by clouds. The polarisation pattern and the MW pattern have some similarities: they are both large, low-contrast structures that will dominate in the sky when they are visible. Examples of these insects include the desert ant *Cataglyphis* [12] and the field cricket *Gryllus campestris* [13], both of which can utilise celestial cues to keep their heading direction when they are walking, even if their movement is disrupted along the yaw axis.

A moonless, clear night sky is significantly dimmer than full daylight, with light intensity dropping from 10,000 lux during the daytime to just 0.0001 lux under such night-time conditions [15]. *Scarabaeus satyrus* is a nocturnal dung beetle living in the savannas of Southern Africa. They have been observed to navigate in straight lines at night while transporting the balls of dung they have painstakingly created away from the source to a suitable location for oviposition and burial. This behaviour is desirable to avoid competition with members of the same species who would benefit from the reduced energy and time use achieved by seizing another individual’s ball. When the moon and lunar sky polarisation pattern are absent, *Scarabaeus satyrus* relies on the MW as a celestial directional cue for the transition of the transit away from the source of the dung [2,16,17,18]. Evidence indicates that they use the MW only as a short-term heading reference. Their studied behavior requires a temporary sense of relative heading direction, and there is no evidence of an MW almanac, clock, or other systems that would indicate that the behaviour is a genuine celestial navigation system. The mechanism could be accurately described as the MW being used as a celestial landmark. However, the distinction between a compass and a landmark is not substantial considering the timeframe and the behavior of the beetle.

### 2.2. The Milky Way

**Figure 2:**
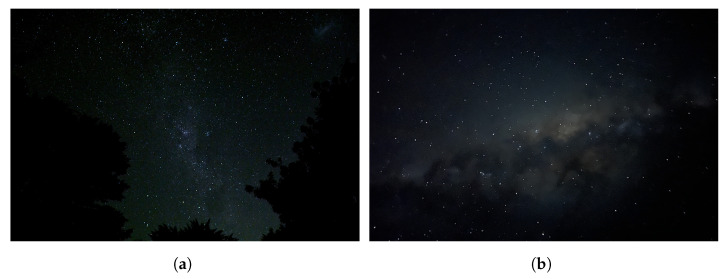
The Milky Way observed under a rural sky in South Australia. (**a**) The Milky Way captured using a DSLR camera and (**b**) the Milky Way captured using a mobile phone.

**Figure 3:**
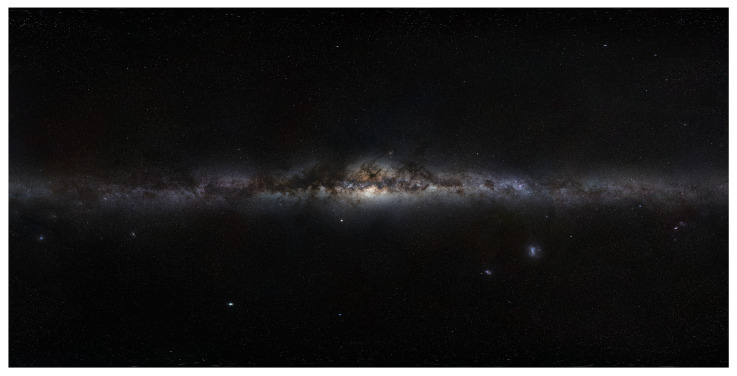
A 360° panoramic picture assembled from several photographs that cover the Milky Way’s northern and southern celestial spheres. Image: Licenced under a Creative Commons Attribution-Share Alike 4.0 International licence by the European Southern Observatory (ESO).

The Milky Way (MW) is a large structure in comparison to other celestial bodies, and a typical image of it is illustrated in Figure 2. The MW is not uniform, and contains variations in brightness across its up to 30° wide span across the Southern night sky. Since the 1920s, astronomers have known that this band represents an edge-on view of the Galaxy from our location inside. Spiral galaxies, like the MW, are pinwheel-shaped and composed of visually dense gas clouds, nebulae, and hundreds of billions of stars [19]. The MW Galaxy has an approximate diameter of 87,400 light-years, and is 1000 light-years wide across the spiral arms. Our solar system is situated in the Orion Spur, approximately 28,000 light-years from the galactic center. The Great Rift, often referred to as the Dark Rift, is the name given to a visually prominent dark structure in the MW, which is seen as dark areas within a luminous band caused by interstellar clouds in cosmic dust, see Figure 3. The MW’s luminosity varies across its span; thus, the visibility of the MW will depend on the time of year and the observer’s location on Earth.

Despite being low in contrast, the MW subtends a much larger visual angle than any other star, constellation, or planet, and its shape is composed of both a high density of stars and a low-spatial-frequency luminous structure. A camera with suitable sensitivity to light needs only a very low angular resolution to detect the MW. We have previously demonstrated, through motion blur filters applied to real and synthetic images, that the imaging of the Milky Way is largely unaffected by motion blur [20].

### 2.3. Celestial Application of Deep Learning

With the development of computer vision technology in recent years, various advanced approaches have been exploited. To date, few studies on deep learning (DL)-based models for celestial navigation have been performed. Fortunately, DL techniques for visual object detection are a fundamental aspect of computer vision and have been widely studied. DL-based object detectors are classified into two categories, one-stage object detectors and two-stage object detectors. A two-stage detector is one that first generates a pre-selected box, called a region proposal (RP), and which then focuses on the region of interest (ROI). Further candidate regions are classified using convolutional neural networks (CNNs). The well-known two-stage detector approaches include RCNN [21], Fast RCNN [22], Faster RCNN [23], Mask RCNN [24], etc. In contrast, one-stage object detector models do not require the use of an RP stage, which is much simpler and more efficient compared to a two-stage object detector. Examples of single-stage models include YOLO (You Only Look Once) [25], SSD [26], DSSD [27], etc. The YOLO algorithm is a CNN-based object detection framework which has been widely explored and implemented in the computer vision field. Various versions of YOLO have been introduced for object recognition, including YOLOv2 [28], YOLOv5 [29], and YOLOv8 [30], etc.

Also, deep learning-based image recognition for navigation is a similar problem that has been attracting a considerable amount of interest. For instance, Pan et al. proposed a deep learning-based fine-grained recognition model named RMA (ResNet-Multiscale-Attention) to achieve visual recognition of different types of navigation marks for sea transportation [31]. Additionally, Wang et al. [32] proposed a visual navigation framework for drogue detection and position tracking to achieve the aerial recovery of unmanned aerial vehicles (UAVs). In addition to Earth observation (EO) navigation applications, there are also some studies in the field of space exploration that use DL-based vision-relative methods for navigation and landing [33,34,35].

### 2.4. Contribution of This Study

This paper proposes an MW detection and orientation algorithm that utilises a deep neural network, YOLOv8, for segmenting the MW shape. This research consists of the following tasks:

- Constructing a Milky Way object detection and image segmentation dataset.
- Accurately localising the MW by using YOLOv8-seg models and calculating the moving angle to determine its orientation.
- Developing a new model that combines the proposed YOLOv8-seg and normalised second central moments to calculate the MW orientation angle. This method addresses situations where the image thresholding method is not applicable for MW region detection, such as on full moon nights or during artificial light interference.

## 3. Methods

Previously, we demonstrated a conventional computer vision method based on object detection and segmentation, with edge detection that could extract the angle of the orientation of the MW [36]. This method was suited to conditions under which the MW could be cleanly segmented in a night sky, sans distractors such as trees and distant glow over cities that would confuse the segmentation process.

We also tested the MW orientation algorithm on both real and synthetic images. We showed that a useful characteristic of the MW as an orientation reference was its visual resilience to motion blur compared to stars. Motion blur is a substantial problem for long-exposure images on moving platforms subject to rotational vibration [20].

**Figure 4:**
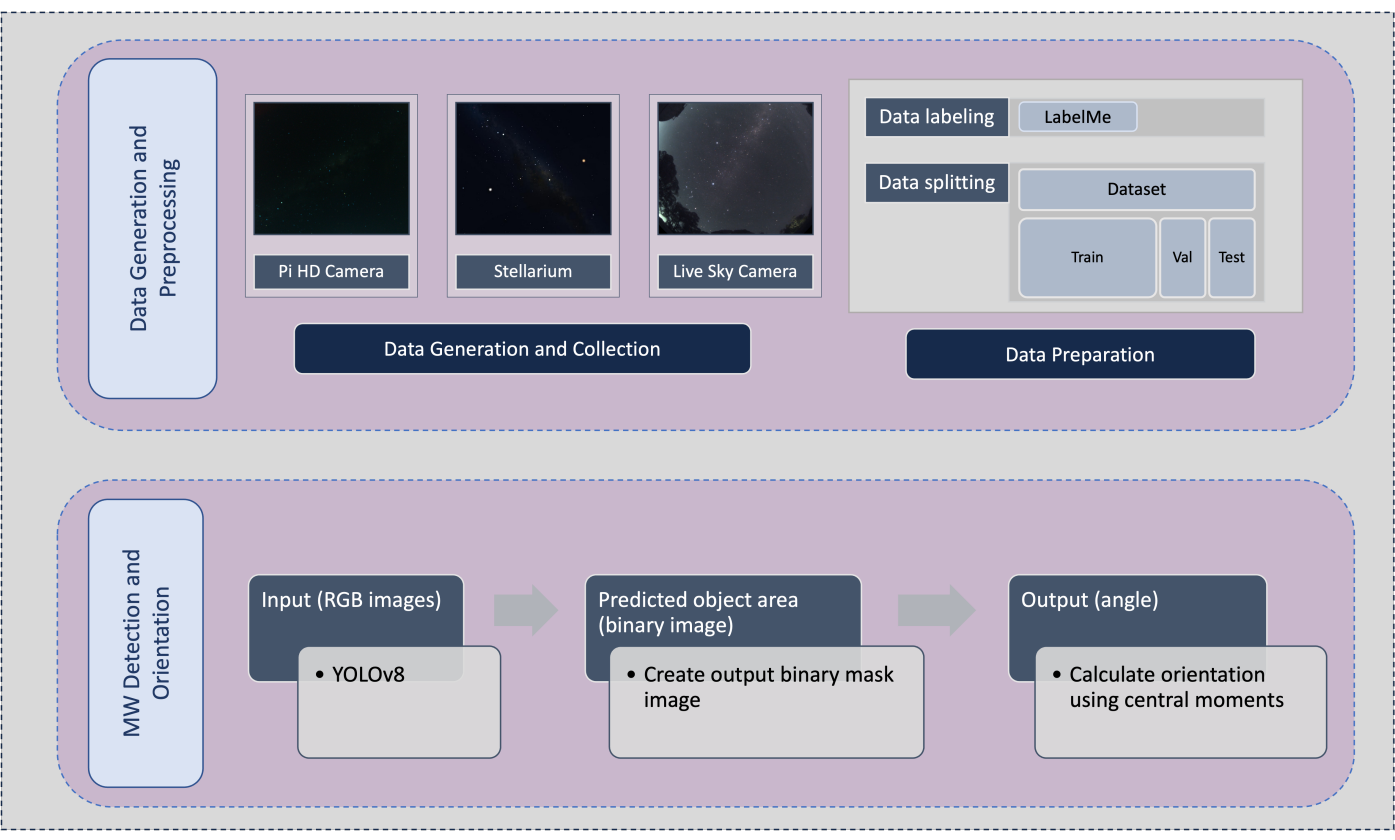
An overview of the proposed methodology.

However, segmentation of the MW from the background is a substantial issue in an environment with multiple light sources, particularly near the horizon. Therefore, in this paper, to reduce the impact of backgrounds (artificial lights, moonlight, etc.) on MW extraction, the YOLOv8 network was proposed for obtaining regions of interest in MW images. This paper explains the use of a deep learning model, YOLOv8, for the purpose of detecting MW, even in conditions such as a full moon night. The aim of this approach is to reduce the image to an angle that represents the orientation of the MW, which might then be input to a navigation system. The proposed method in this paper, the MW orientation algorithm (MWOA), comprises the steps illustrated in Figure 4.

- Data generation and collection: The dataset utilised for training purposes is explained in Section 4.
- Model selection, training, and evaluation: Upon obtaining and labelling the images, the YOLOv8 model is trained on the prepared dataset. The trained model’s performance is evaluated in metrics, which provide a measure of how well the model performs in MW detection, in Section 5.1.1.
- Orientation Estimation: This step deploys the trained model to process the test images, generates the predicted MW binary images, and calculates the angle.

### 3.1. YOLOv8 Model

YOLOv8 [30] is capable of handling multiple vision tasks, including object detection, segmentation, pose estimation, tracking, and classification. YOLOv8 is presented in five different model sizes: YOLOv8n (nano), YOLOv8s (small), YOLOv8m (medium), YOLOv8l (large), and YOLOv8x (extra-large). The backbone of YOLOv8 is similar to YOLOv5, with some changes: the C3 module is replaced with a C2f module, and the head part is changed to a decoupled head structure. Moreover, the YOLOv8 model changes from an anchor-based to an anchor-free model, which decreases the number of box predictions and increases the Non-Maximum Suppression (NMS). YOLOv8 also provides an instance semantic segmentation model, which is a step further than the object detection model called YOLOv8-Seg. There are five YOLOv8 segmentation models: YOLOv8n-seg, YOLOv8s-seg, YOLOv8m-seg, YOLOv8l-seg, and YOLOv8x-seg. In this paper, experiments were conducted on YOLOv8s-seg and YOLOv8m-seg to find the Milky Way and extract the MW’s shape.

### 3.2. Orientation Estimation

Once the Milky Way area in the night sky images has been predicted by using the proposed YOLOv8-seg model, an object mask (binary image I(x,y)) can been generated; then, the normalised second central moments are utilised to calculate angular information for the extracted area of interest in the MW region. The coordinates of the centroid ($\bar{x},\bar{y}$) are determined by finding the mean of the pixel coordinates in the *x* and *y* directions, respectively.

(1) $$\begin{array}{c} \bar{x}=\frac{1}{N}\sum_{i=1}^{N}x_{i} \end{array}$$

(2) $$\begin{array}{c} \bar{y}=\frac{1}{N}\sum_{i=1}^{N}y_{i} \end{array}$$

where *N* represents the total number of pixels within the region, while $x_{i}$ and $y_{i}$ indicate the *x* and *y* coordinates of the *i*-th pixel within the region.

The MW orientation is obtained using the calculated second-order central moments. The central moment (μ) represents the coordinates of the mean. The normalised second central moments for the region ($\mu_{xx},\mu_{yy},\mu_{xy}$) can be computed as follows:

(3) $$\mu_{xx}=\frac{\sum_{i}x_{i}^{2}}{N}+\frac{1}{12}$$

(4) $$\begin{array}{c} \mu_{yy}=\frac{\sum_{i}y_{i}^{2}}{N}+\frac{1}{12} \end{array}$$

(5) $$\mu_{xy}=\frac{\sum_{i}x_{i}y_{i}}{N}$$

where the number of pixels in the region is denoted by *N*, and *x* and *y* represent the pixel coordinates within the region relative to the centroid. The normalised second central moment of a pixel with unit length is represented by $\frac{1}{12}$

(6) $$\begin{array}{cc} \; & \mathrm{num}=\left\{\begin{array}{cc} \mu_{yy}-\mu_{xx}+\sqrt{{\left(\mu_{yy}-\mu_{xx}\right)}^{2}+4\mu_{xy}^{2}}, & \;\mathrm{if}\;\mu_{yy}>\mu_{xx}\\ 2\mu_{xy}, & \;\mathrm{otherwise}\; \end{array}\right. \end{array}$$

(7) $$\begin{array}{cc} \; & \mathrm{den}=\left\{\begin{array}{cc} 2\mu_{xy}, & \;\mathrm{if}\;\mu_{yy}>\mu_{xx}\\ \mu_{xx}-\mu_{yy}+\sqrt{{\left(\mu_{xx}-\mu_{yy}\right)}^{2}+4\mu_{xy}^{2}}, & \;\mathrm{otherwise}\; \end{array}\right. \end{array}$$

For the orientation of the MW region, Orientation is the direction (angle) calculated based on the normalised second central moments of the region (MW shape).

(8) $$\begin{array}{c} \;\mathrm{Orientation}\;=\frac{180}{\pi }\mathrm{atan}\left(\frac{\;\mathrm{num}\;}{\;\mathrm{den}\;}\right) \end{array}$$

## 4. Data Collection

**Figure 5:**
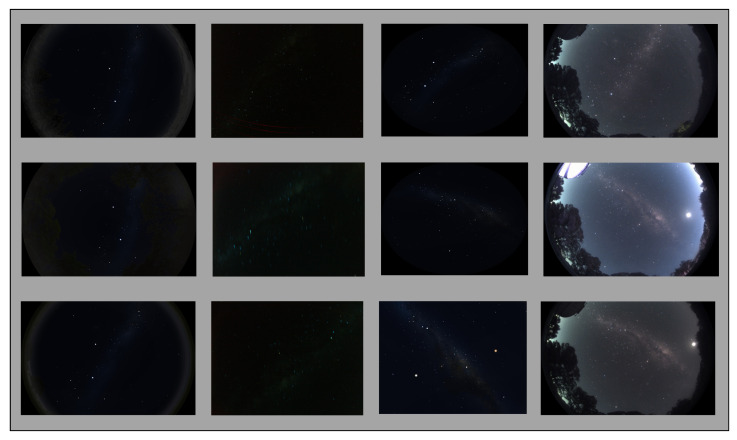
Some images from the dataset used: 1st column: Synthesised night sky images with surroundings; 2nd column: Mallala night real sky images; 3rd columns: Stellarium sky images; 4th column: Mount Burnett Observatory live sky camera images.

Preparing an appropriate dataset is an important factor for ensuring the accuracy of deep learning model performance. Our dataset contains a diverse range of night images containing the MW, including synthetic images, real night images captured by a car-based PI HD camera, and real night sky images from an all sky camera [37]. The dataset contains 735 images. Figure 5 shows some images from the dataset.

### 4.1. Synthetic Generation of Images

The MW’s visibility is limited in some regions due to anthropogenic light, which is also a problem for optical astronomy in these locations. Also, capturing good night sky images with proper exposure to show the Milky Way clearly is quite a challenge [38]. To address this data collection issue, we utilised Stellarium (version 0.22.2) to produce some simulated MW images.

The open-source desktop planetarium software Stellarium was developed by enthusiasts to simulate the specific celestial sphere according to the entered time and location parameters [39]. The following configurations in Stellarium were used in this study: date, position, Milky Way brightness/saturation, light pollution level (LP), and more. The light pollution levels LP3 (rural sky) and LP4 (rural/suburban transition) were chosen when generating the synthetic MW images [40]. The default MW brightness/saturation configuration (brightness: 1, saturation: 1) was applied to each simulated test image. This software also provided MW shape information by increasing the brightness/saturation of the MW area, and can be used as a reference to determine and annotate the MW in real night images which always have low contrast and low intensity. In order to prepare a diverse deep learning dataset, simulated images with different sky parameter settings were used, and the dataset also contained night images with a range of landscapes.

### 4.2. Field Image Acquisition

**Figure 6:**
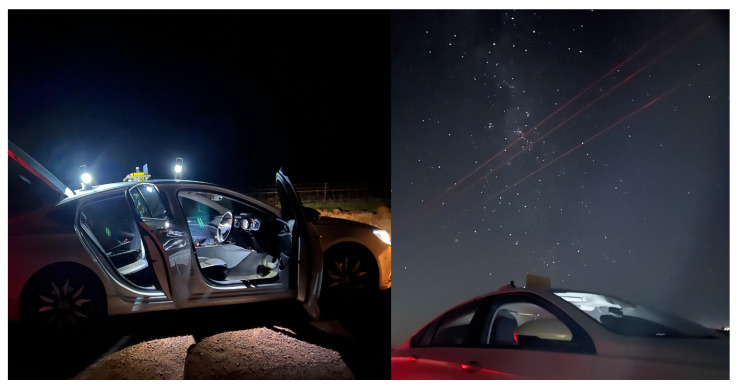
Car-based image acquisition system.

Data acquisition was achieved using a system that included a single-board computer (SBC) for storage, triggering, and networking to a cloud server, and a standard OEM camera, which was installed on the roof of the test vehicle, as illustrated in Figure 6. A field trial was undertaken to obtain a dataset of real night sky images by driving within a chosen low-population-density area, located among the wheat fields near Mallala, South Australia. The image capture equipment was a Raspberry Pi HD camera coupled with a 6 mm wide-angle camera lens (CS-Mount). The initial images were taken when the vehicle was not moving, but with the engine running, leading to an inevitable amount of motion. After we had acquired enough stationary images, we proceeded to gather a set of moving images by driving the vehicle at around 40 km/h. This allowed us to test the optical equipment under both engine vibrations, which are high-frequency, and road vibrations, which are lower-frequency. The vehicle’s route included a series of 90-degree turns and mostly consisted of unpaved roads. More images were taken while the vehicle was not in motion and the engine was turned off. This helped to gather reference data without significant motion blur. Further details are included in Table 1. All images within the dataset were taken in high definition (3280×2464 pixels) and with exposure times of 10 s, 20 s, and 30 s.

**Figure 7:**
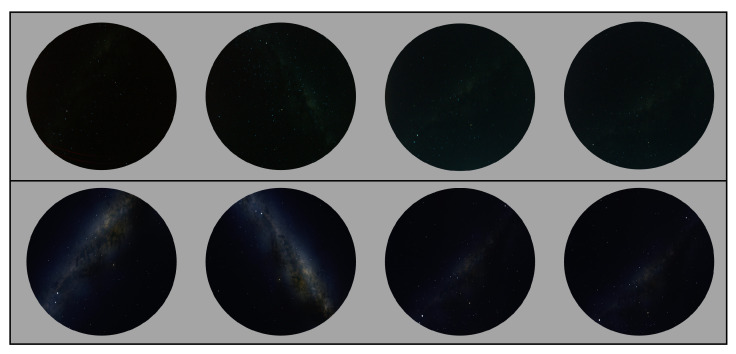
**Top row**: real night sky images captured from the Mallala trip; **bottom row**: the simulated sky images were generated when the same date and location were selected and the MW brightness configuration was increased (Stellarium setting: MW brightness = 3, LP = 4).

Figure 7 shows some of the real night images in our dataset and some simulated images taken at the same location, date, and time with the real night images of the MW area, but with more brightness, which is adjustable. The simulated images can be used as reference images for dataset annotation.

### 4.3. Live Sky Camera

**Figure 8:**
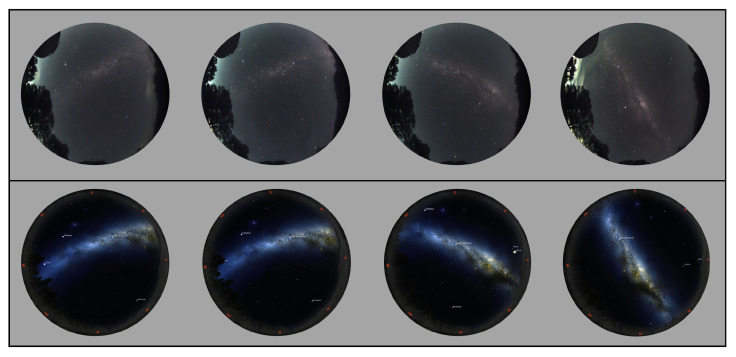
**Top row**: the real night sky images captured from Mount Burnett Observatory; **bottom row**: the simulated sky images were generated when the same date and location was selected and the MW brightness configuration was increased (Stellarium setting: MW brightness = 5, LP = 4).

Additional real night sky images included in the dataset were sourced from a live sky camera situated at Mount Burnett Observatory in Australia [37], the all sky camera. Table 2 provides further details regarding the live sky camera. Figure 8 shows some of the real night sky images captured from Mount Burnett Observatory used in our dataset, and some simulated images taken at the same location, date, and time but with increased MW area brightness, which is adjustable.

### 4.4. Image Annotation

**Figure 9:**
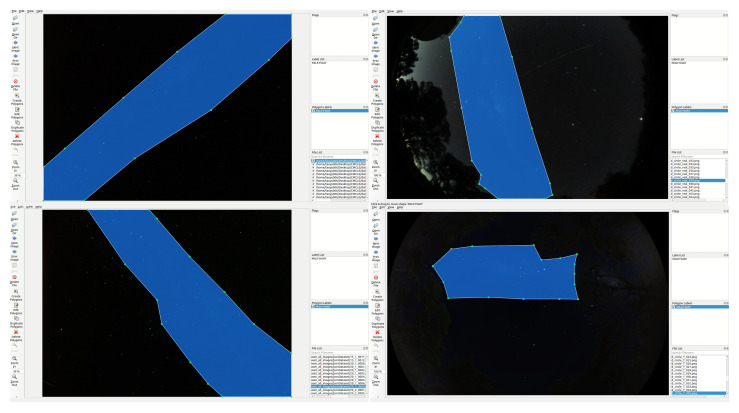
Example annotation images of Milky Way object detection and instance segmentation. The bottom-right image is a synthetic image with a landscape background, and part of the MW is hidden by trees.

The dataset was manually annotated with LabelMe (4.5.7) software. During the label task for segmentation, the location of the MW area was manually marked in polygon regions, with each polygon consisting of 10–30 points, as shown in Figure 9. The LabelMe annotation tool generates a .json file for each image. The difficult point in the annotation processing is that the night sky images are dim and low-contrast, which means the MW shape is hard to annotate.

Since the transitional edge between the MW and the sky area is hard to distinguish, the annotation was performed by only one person to maintain consistency. We used simulated images to ensure the accuracy of our annotations. By using Stellarium, we could set the same date and location as in the dataset image, and then compare the simulated image to determine the MW shape. The increased brightness of the MW area in the simulated images was often used as a reference while annotating. However, manual labelling of the MW shape can affect accuracy. As mentioned above, Figure 7 shows some examples of real night images and simulated images in our dataset.

The dataset was then divided into training, validation, and test sets at a ratio of 7:1.5:1.5. Table 3 provides the details of these sets. The format for the object detection and segmentation datasets was YOLO.

## 5. Results

### 5.1. Milky Way Detection Using YOLOv8

A YOLOv8-seg model was trained on our MW dataset using transfer learning. We initialised the segmentation models with pre-trained weights on the COCO-Seg dataset, which included 80 pre-trained classes, and fine-tuned it on our MW dataset. Table 4 shows the parameters of YOLOv8m-seg used during the training of the model for detecting the Milky Way.

#### 5.1.1. Object Detection Evaluation

**Figure 10:**
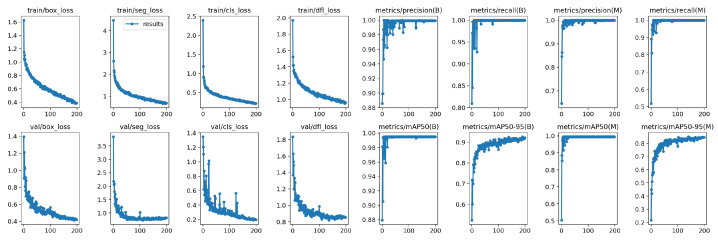
A loss map of the proposed model (YOLOv8m-seg).

To quantitatively evaluate the performance of the proposed object detection approach, the evaluation criteria (*recall*, *precision*, and the mean Average Precision (*mAP*)) were calculated as follows:

(9) $$Recall=\frac{TP}{\left(TP+FN\right)}.$$

(10) $$Precision=\frac{TP}{\left(TP+FP\right)}.$$

(11) $$AP=\int_{0}^{1}P_{i}\left(r_{i}\right)d\left(r_{i}\right)$$

(12) $$mAP=\frac{\sum_{i=1}^{N}AP}{N}$$

where *TP* is the true positive, *FP* is the false positive, and *FN* is the false negative. Average Precision (*AP*) was calculated for each class. *N* is the total number of classes. $P_{i}\left(r_{i}\right)$ is the precision at recall $r_{i}$. A loss map of the proposed YOLOv8 segmentation model is shown in Figure 10.

(13) $$FM=2\cdot \frac{Recall\times Precision}{\left(Recall+Precision\right)}.$$

**Figure 11:**
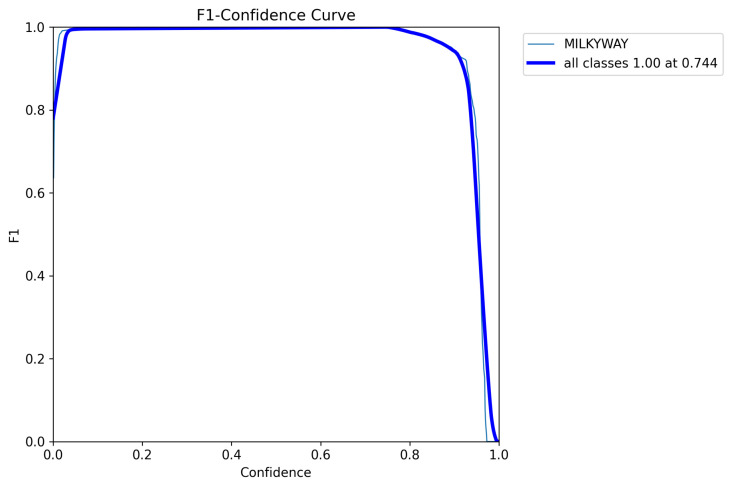
A MaskF1 curve of the proposed model (YOLOv8m-seg).

**Figure 12:**
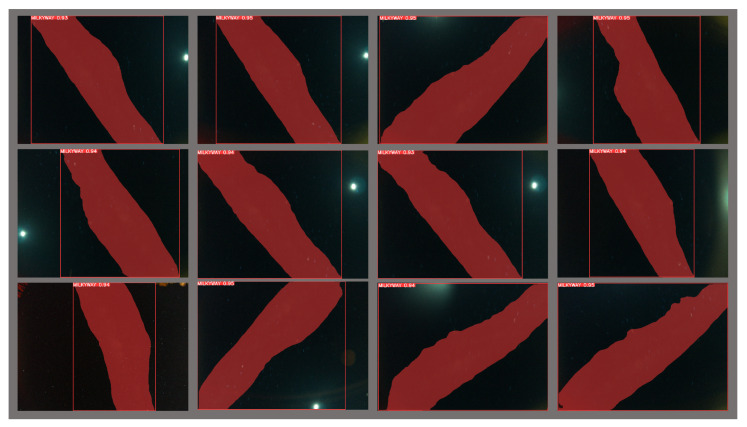
The result of the proposed model for MW detection.

The *FM* score is defined as the harmonic mean (F-Measure) of the *precision* and *recall* values, which measure the matching weight for the predicted observations and the ground truth positives. A Mask F1 curve of the proposed YOLOv8m-seg model is shown in Figure 11. Figure 12 presents MW detection results with the proposed model for some field images.

Considering the different sizes of the YOLOv8 segmentation models, a comparison with some models of other sizes is shown in Table 5.

Table 6 shows the results of training different sized YOLOv8-seg models with the dataset containing 500 simulated night sky images, and Table 7 shows the results of training the YOLOv8m-seg model with the dataset that includes 200 simulated night sky images, 100 all sky camera night images, and 135 Mallala trip night sky images.

#### 5.1.2. Error/Invalid Results

**Figure 13:**
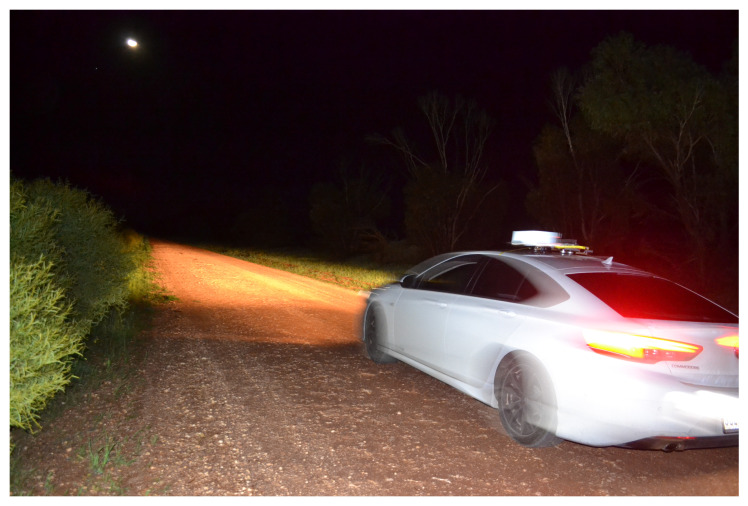
Vehicle-based data acquisition system on mission.

Under low-light conditions, like the night time in rural areas, extending the exposure time is a way to capture sufficient light, but it simultaneously exacerbates the amount of blur. Figure 13 was taken from the vehicle-based data acquisition system in Mallala. As we can see, the moon and the car light were interfering light sources.

**Figure 14:**
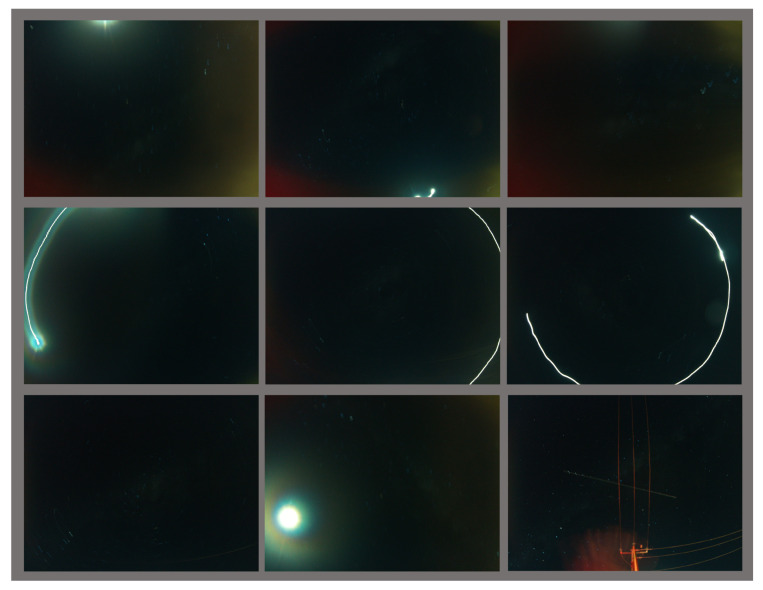
Some invalid images. **Top row**: bright moon and car lights; **second row**: high blur and car rotating; **third row**: some other types of invalid images.

Therefore, during MW image capture, there may be some images with noise that are not valid for MW detection. Figure 14 shows some invalid night sky images captured during the trial. All of these images could not be used for MW detection due to disruptions in image details caused by hardware and environmental conditions. For example, the first row of Figure 14 demonstrates images impacted by car lights and moonlight. In the second row, when the car turns, the moon can be seen as a bright curved light due to the long camera exposure times. The third row shows additional images affected by various disturbances.

**Figure 15:**
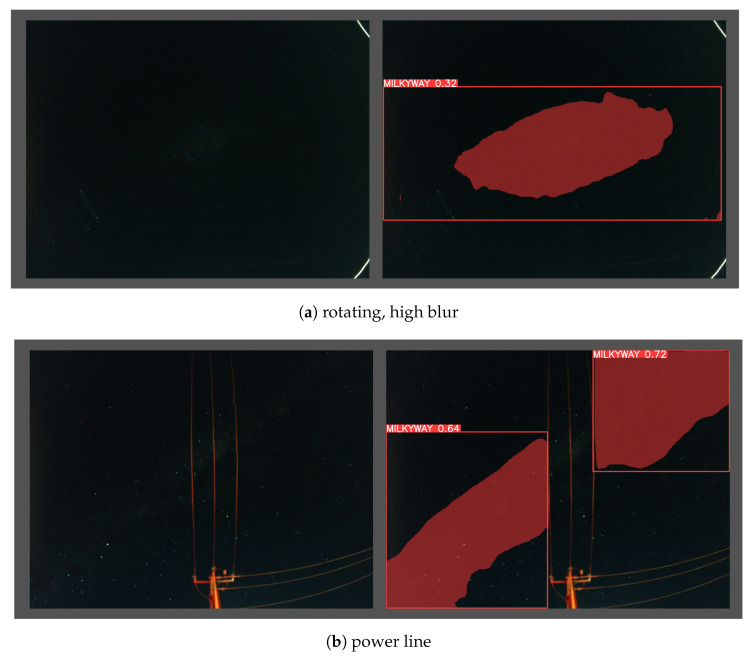
Examples of error results.

Figure 15 illustrates two images that cannot be used for angular calculations. Because of the long exposure time, Figure 15a was captured during rotation, which caused vibration and blur effects in MW detection. Figure 15b shows the predictive MW region, which is affected and divided by the power line.

### 5.2. Angle Calculation

**Figure 16:**
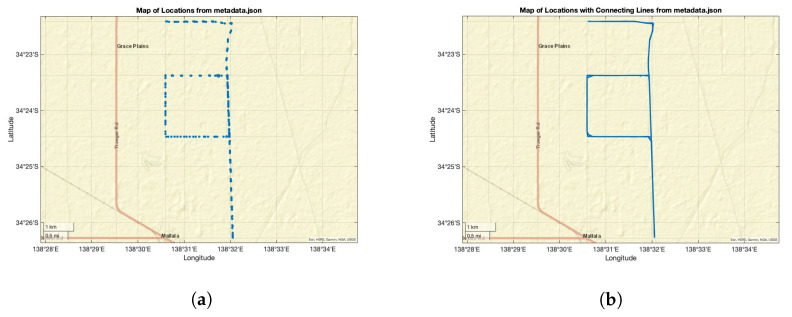
GPS map. (**a**) Map of locations with points from metadata and (**b**) map of locations with connecting line.

Figure 16 shows the GPS locations from the metadata while the night sky images were acquired; the trip duration was around 3 h.

#### Quantitative Comparison

**Figure 17:**
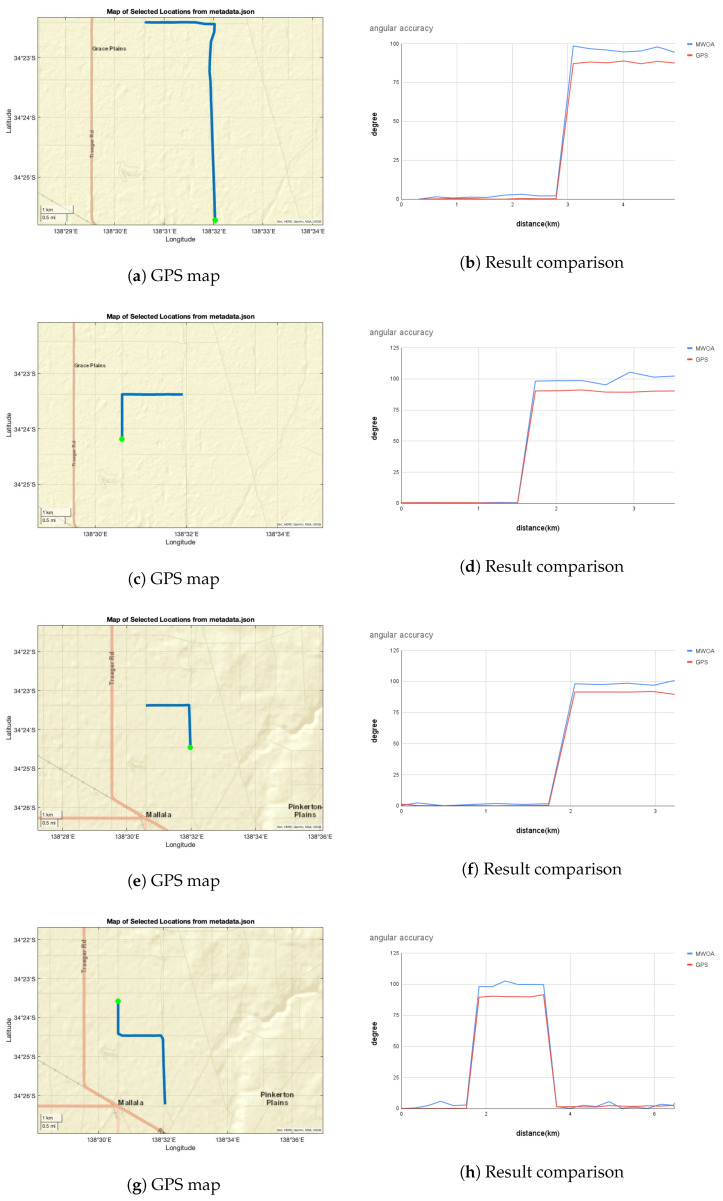
Angular accuracy calculated with MWOA for certain sections of the road. The green coloured dot on the GPS maps indicates the starting point.

Figure 17 shows the angular change comparison results between the proposed method, MW orientation algorithm (MWOA), and GPS data (GT) for the trial in Mallala on 25 July 2023. In order to show the comparison results in a short distance, we separated the whole trip dataset, which was obtained over around 3 h, into segments, each sub-dataset containing at least one 90° rotation.

In Figure 17a, the GPS route from the metadata is illustrated, the green dot indicating the starting point and the blue line denoting the driving route, which were selected between the start time of 8:28 p.m. and the end time of 8:45 p.m. Figure 17b shows the angular change comparison results between the proposed method (blue line) and the ground truth (red line).

The ground truth orientation angles calculated from the GPS data closely align with the angles calculated from the MWOA. We observed a difference of roughly 5–10° between the proposed method and GT for both left and right turns at certain locations. At some points along the road, the maximum amplitude difference between angles becomes roughly 10°. However, the MWOA responds well to orientation changes and direction calculations along straight paths. Angle differences may occur for various reasons, such as light pollution caused by car lights and moonlight, as well as the impact of motion blur on image details. This observation is clearly visible in the graphs, with the starting point indicated on the map. The deviations in MWOA angular accuracy might be due to limitations in the hardware and long exposure time, which might cause the MW region detection to not be same, leading to errors during dynamic movement.

**Figure 18:**
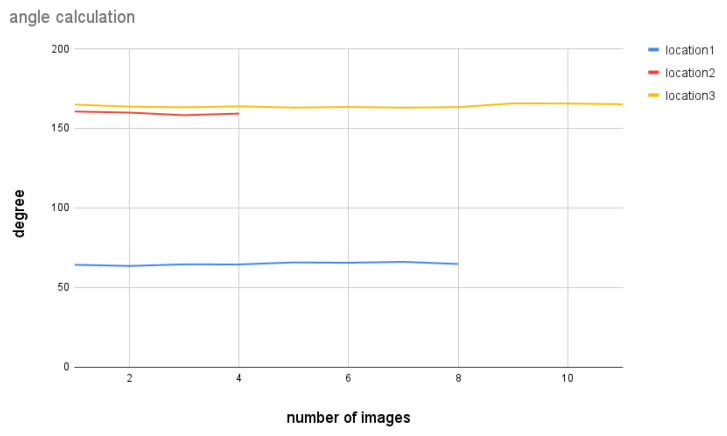
Angle calculation for certain locations of the map while the vehicle is not moving.

Figure 18 shows the result when the car is not moving. The lines indicate three different locations during the trip with stationary periods. The results imply that the MWOA provides reliable MW detection and direction information when there is not much disturbance caused by movement; even the moonlight and car lights are presented in this real night scenario.

## 6. Discussion

The hardware used for this study was essentially hobby-grade. This caused long exposure times and substantial optical distortion. It would be useful to capture more imagery; however, the MW is not visible everywhere. The MW is best viewed far from population centres, creating challenges when attempting to capture enough data for deep learning training datasets. There are also a range of different atmospheric conditions that would ideally be photographed for training data. Complete coverage of the space of all phenomena would require thousands of images from unique scenarios. This would be an exorbitant use of resources, showing why deep learning solutions are at their best when a dataset has been created and classified by a large number of observers. In this study, the simulated generation of data appears to have performed as envisaged, leading to considerations of what might be involved in adding atmospheric effects and terrain features to the simulator’s output.

Some examples of changes in the detected shape of the MW when the car is turning can be seen in Figure 15a, which presents an image affected by this scenario. Tall and overhead infrastructure at the site interfere with the boundary of the MW. Figure 15b shows the predicted MW region, which was affected by the power lines. It is notable that the well-demonstrated terrestrial animal model for MW orientation, the dung beetle, lives in treeless and relatively flat environments. Many of these problems would not exist in the aerial domain, and none of these problems would exist at high altitude.

The MW object in the night sky does not have a distinct brightness boundary with the sky, as it fades away at the edges. Therefore, annotation of the MW shape was relative to the annotator’s judgment. It is hard to annotate the exact shape of the MW due its smoky shape. To maintain consistency in annotation, the annotations were carried out by one person.

The hardware setup used for the data capture was mounted on a car. The vibration and the movement of the car might have interfered with the image stability, adding motion blur noise to the images. The headlights of the car were set to the lowest beam angle during image capture. However, the reflected light seemed to appear in some captured images. The addition of dust and mist provides material for the scattering of light from below the sensor horizon down into the camera. Most of the images were unaffected by the car light, but some were severely affected (e.g., Figure 13). We manually removed these images from our dataset during the data preprocessing stage. When the car was turning, the other bright objects in the sky, such as the moon and bright stars, created curved light trajectories on the images, making them unusable for the experiments.

This method has not been developed or tested for partial occlusion, and has not been engineered for graceful degradation when the MW is partially or fully occluded. The dung beetle has exhibited a number of orientation behaviours under different circumstances, including a lunar reference and the Milky Way, in various combinations of conditions [1,17]. In exoatmospheric and high-altitude environments, the MW is reliably present. On Earth, it is one of many possible direction cues, subtracting moonless nights from the range of conditions in which celestial orientation is not possible.

## 7. Conclusions

In this work, an MW shape detection algorithm based on YOLOv8m-seg was used to reduce the interference of other light sources in the MW compass task. The YOLOv8m-Seg network with our own training dataset achieved a segment mAP@0.5 of 84.7% on the validation set. Combined with the proposed YOLOv8m-seg model and angle calculation method, the new model effectively reduced the influence of complex backgrounds. The moving angle calculation comparison with the GT data from the field trial in Mallala shows that for short-term navigation, the segmented MW image can be used as a reliable orientation cue. Future research will continue to optimise the YOLOv8m-Seg model to improve its segmentation accuracy for MW detection.

## Figures and Tables

**Table 1:** Information about the data acquisition system, Mallala, South Australia.

Items	Specification
Location	Mallala, South Australia, Australia
Speed	0–40 km/h
Camera	Pi HD camera
Exposure	10–30 s
Computer	Raspberry Pi 4B

**Table 2:** Information about the all sky camera.

Item	Specification
Location	Mt Burnett, Victoria, Australia
Latitude	37.9725 S
Longitude	145.4955 E
Camera	ASI224MC
Exposure	30 s
Computer	Raspberry Pi 3B+

**Table 3:** MW object detection and segmentation dataset construction.

Dataset	Ratio	Number	Dataset Format (Segmentation)
Training set	7	513	YOLO
Validation set	1.5	111	YOLO
Test set	1.5	111	YOLO

**Table 4:** The hyperparameters for training the MW detection method.

Training Hyperparameters	Details
Epoch	200
Image size	640
Batch size	8
Learning rate	0.01

**Table 5:** A comparison of different scales and parameters of YOLOv8-Seg models.

Model	Weights	Batch	Epoch	Mask (mAP0.5)	Mask (mAP@0.5:0.95)
YOLOv8	YOLOv8m-seg	8	100	0.995	0.847
	YOLOv8m-seg	8	200	0.995	0.847
	YOLOv8s-seg	8	100	0.995	0.852
	YOLOv8s-seg	8	200	0.995	0.853
	YOLOv8s-seg	12	200	0.995	0.852

**Table 6:** The results of training the YOLOv8-seg model; dataset: all simulated (500).

Model	Weights	Batch	Epoch	Mask (mAP0.5)	Mask (mAP@0.5:0.95)
YOLOv8	YOLOv8m-seg	8	100	0.995	0.841
	YOLOv8m-seg	8	200	0.995	0.844
	YOLOv8m-seg	8	300	0.995	0.839
	YOLOv8s-seg	12	100	0.995	0.838

**Table 7:** The results of training the YOLOv8-seg model; dataset: simulated (200) + all sky camera (100) + Mallala (135).

Model	Weights	Batch	Epoch	Mask (mAP0.5)	Mask (mAP@0.5:0.95)
YOLOv8	YOLOv8m-seg	8	100	0.995	0.85
	YOLOv8m-seg	8	200	0.995	0.863

## Data Availability

The original contributions presented in this study are included in the article. Further inquiries can be directed to the corresponding author(s).

## Abbreviations

The following abbreviations are used in this manuscript:

MW	Milky Way
ESO	European Southern Observatory
DL	deep learning
ROI	region of interest
RP	region proposal
CNNs	convolutional neural networks
YOLO	You Only Look Once
RMA	ResNet-Multiscale-Attention
UAV	unmanned aerial vehicle
EO	Earth observation
MWOA	Milky Way orientation algorithm
NMS	Non-Maximum Suppression
LP	light pollution
SBC	single-board computer
mAP	mean Average Precision
TP	true positive
FP	false positive
AP	Average Precision
GT	ground truth
